# Intracapsular Osteochondroma of the Proximal Humerus Causing Restriction of Shoulder Joint Movements: An Uncommon Entity

**DOI:** 10.7759/cureus.110766

**Published:** 2026-06-13

**Authors:** Chandrashekar Patil, Manisha Vishwanath, Nandakishore G Patil, Suresh Chevagoni, Paras Gupta

**Affiliations:** 1 Radiology, Mallareddy Medical College for Women, Hyderabad, IND; 2 Radiology, Hubli Scan Centre, Hubli, IND; 3 Radiology, Malla Reddy Vishwavidyapeeth, Hyderabad, IND; 4 Radiodiagnosis, Mallareddy Medical College for Women, Hyderabad, IND

**Keywords:** bursa, cartilage cap, mri, osteochondroma, proximal humerus

## Abstract

Osteochondroma, also termed osteocartilaginous exostosis, is the most common benign bone tumour of all benign osseous neoplasms. These lesions typically arise from the metaphyseal region and project away from the adjacent joint. However, intracapsular or intra-articular osteochondromas are rare, especially in the proximal humerus.

Here, we report 2 cases of intracapsular osteochondroma of the proximal humerus in a 49-year-old woman and a 14-year-old boy, both with shoulder pain and restricted joint movements. The anteroposterior X-ray and magnetic resonance imaging of the shoulder joint demonstrated a bony lesion with medullary continuity with the parent bone and delineated a thin cartilaginous cap. MRI further enabled precise assessment of the lesion’s intra-articular location and cartilage thickness. These cases highlight the emphasis on considering the possibility of intracapsular osteochondroma in the differential diagnosis of unexplained shoulder pain and restricted range of motion. A thorough imaging evaluation, particularly with MRI, is essential for accurate diagnosis, differentiation from other intra-articular pathologies, and appropriate management planning.

## Introduction

Osteochondromas are benign bone lesions characterized by bony outgrowth with cortical and medullary continuity to the parent bone and a cartilaginous cap on their surface [1]. They commonly occur at the metaphyseal region of long bones, particularly around the knee and proximal humerus, typically projecting away from the adjacent joint [2].

An intracapsular or intra-articular osteochondroma is extremely rare in the proximal humerus. When present, it may cause mechanical impingement, joint stiffness, and restriction of movements such as forward elevation and abduction, rather than the palpable mass commonly seen in metaphyseal osteochondromas [3]. Diagnosis is primarily based on imaging findings, as clinical findings may be nonspecific.

Magnetic resonance imaging (MRI) serves as the investigation of choice to differentiate from other cartilaginous tumors, delineate the cartilage cap, its relation to the joint capsule and surrounding soft tissues, and malignant transformation. The MRI protocol for the shoulder joint includes T1- and T2-weighted sequences, fat-saturated proton density (PDFS), or short tau inversion recovery sequence (STIR) in coronal, sagittal, and axial planes with a 3 mm gap and a 3 mm slice thickness diffusion-weighted sequence (DWI) for suspected tumours. Use of intravenous Gadolinium depends on the nature of the pathology, as an optional post-contrast fat-saturated T1 sequence. Here, along with a review of the literature, we report two cases--a 49-year-old female and a 14-year-old boy, both of whom presented with shoulder pain and restriction of joint movements.

## Case presentation

Case 1

A 49-year-old female presented with an insidious onset of pain and progressive restriction of left shoulder movements over six months. There was no history of trauma, fever, or systemic illness. On clinical examination, active and passive abduction and external rotation were significantly restricted, with mild tenderness over the anterior shoulder. No palpable swelling was noted.

An anteroposterior X-ray radiograph of the left shoulder revealed a well-defined, sessile osseous outgrowth projecting from the posteromedial aspect of the humeral head. The glenohumeral joint space appeared maintained.

**Figure 1:**
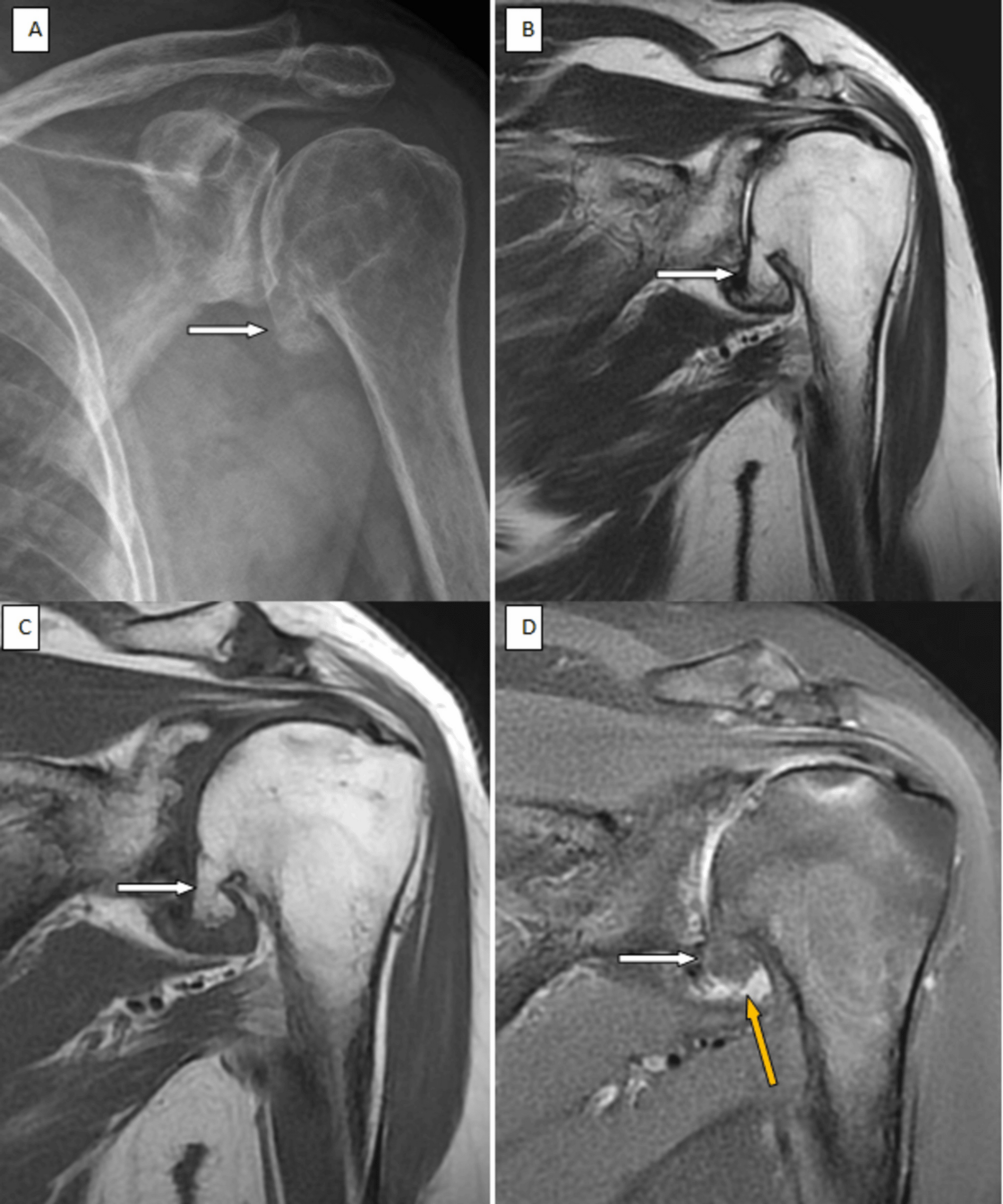
A: X-ray AP view of the left shoulder showing a hanging bony lesion from the humerus (white arrow). B: T2WI and C: T1WI showing an intracapsular bony lesion arising from the posteromedial part of the left humeral head in continuity with the cortex and medulla of the humerus, suggestive of intracapsular osteochondroma (white arrow). D: STIR coronal image notes that the joint capsule is distended, with small effusion (orange arrow). There is no significant thickening of the overlying cartilaginous cap (3 mm). AP: anteroposterior; T2WI: T2-weighted imaging; T1WI: T1-weighted imaging; STIR: short tau inversion recovery sequence

The MRI of the left shoulder (Figure 1) demonstrated a bony outgrowth arising from the posteromedial humeral head, extending into the joint capsule and impinging on the inferior glenoid labrum. The lesion exhibited cortical and medullary continuity with the humeral head. The overlying cartilage cap measured approximately 3mm in thickness on T2-weighted images and showed no abnormal enhancement. There is edema seen in the cartilaginous cap in the inferior axillary recess with central fluid, suggestive of bursitis. There was mild reactive synovitis but no marrow edema or features of malignant transformation. Imaging findings were suggestive of intracapsular osteochondroma.

The patient was admitted to our hospital, and conservative management was done with analgesics, and she was advised to undergo excision of the tumor by the deltopectoral approach. However, the patient was not willing to undergo surgical management.

At the three-week follow-up, the patient was doing better symptomatically; however, there was persistent restriction of movement in the shoulder joint. The patient was advised to undergo surgical excision of the tumor as definitive management.

Case 2

A 14-year-old boy came with complaints of pain in the right shoulder with restriction of shoulder movements on the right side for 2 years; the pain had mildly increased over the last few months. There was no history of trauma, fever, or systemic illness. On clinical examination, active and passive abduction, as well as external rotation, were significantly restricted. No palpable swelling was noted.

An anteroposterior X-ray radiograph of the right shoulder revealed a well-defined, sessile osseous outgrowth projecting from the posteromedial aspect of the right humeral head. The glenohumeral joint space appeared maintained.

**Figure 2:**
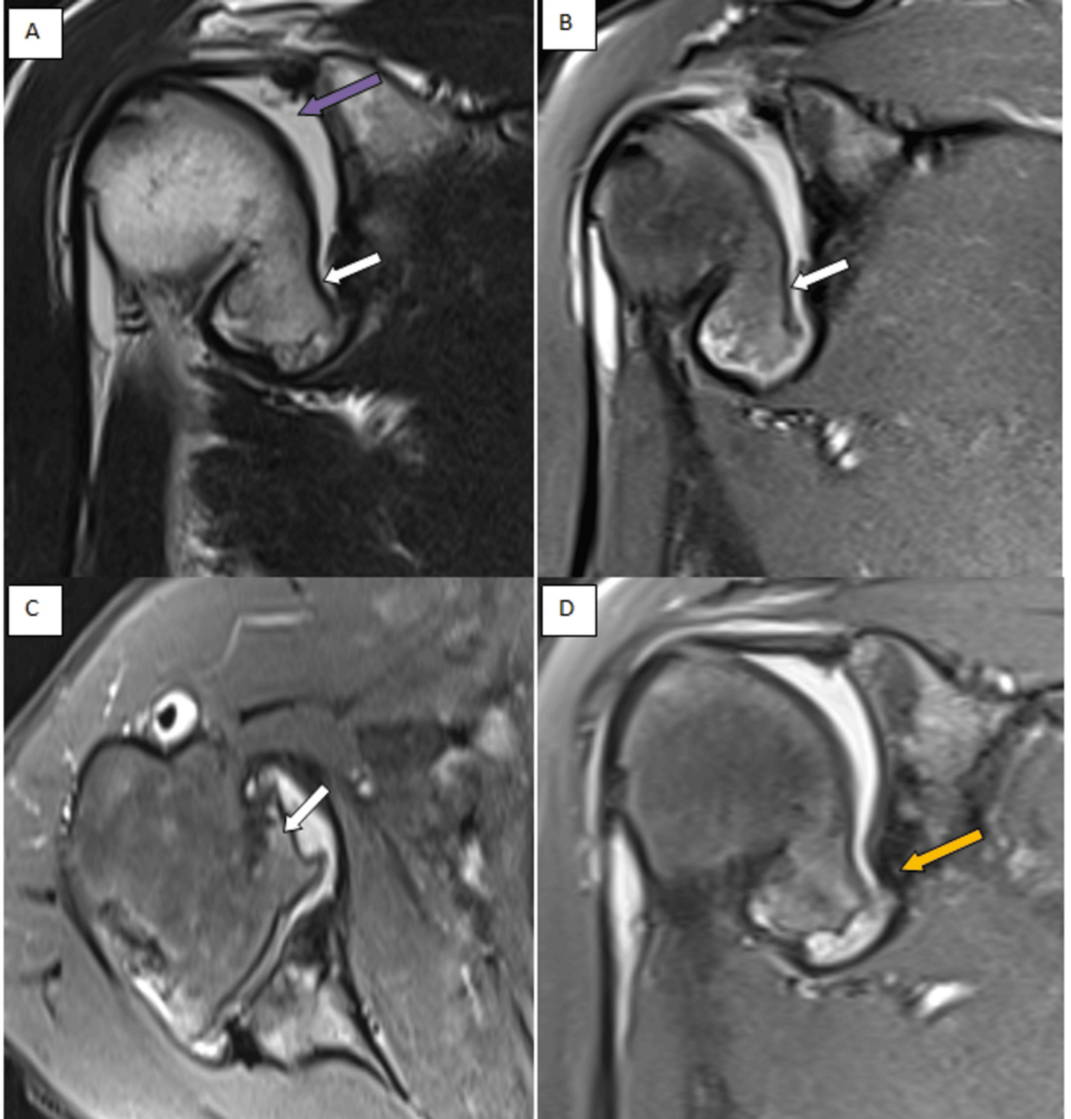
A and B: T2 and short tau inversion recovery (STIR) images showing an exophytic bony lesion arising from the medial part of the right humeral head in continuity with the medulla of the parent bone, consistent with intracapsular osteochondroma (white arrows). Note the mild thickening with distension of the joint capsule and small effusion (purple arrow in figure A). C: Axial view demonstrating the same finding. D: Note osteochondroma impinging on the inferior glenoid (orange arrow), causing pain and restriction of shoulder movements.

On MRI of the right shoulder (Figure 2), T2 and STIR images demonstrated an exophytic bony lesion arising from the medial part of the right humeral head in continuity with the medulla of the parent bone, consistent with intracapsular osteochondroma with mild thickening, with distension of the joint capsule with small effusion. Imaging findings are suggestive of intracapsular osteochondroma.

The patient was admitted to our hospital, conservative management was done with analgesics, and he was advised to undergo excision of the tumor by the deltopectoral approach. However, he was not willing to undergo surgical management.

At the three-week follow-up, the patient was doing better symptomatically; however, there was persistent restriction of movement of the shoulder joint. The patient was advised to undergo surgical excision of the tumor as definitive management.

## Discussion

Osteochondromas typically arise from the metaphyseal region and extend away from the joint; intracapsular variants are extremely rare. Only a handful of cases involving the proximal humerus have been reported in the literature [4]. The intracapsular location often leads to atypical clinical manifestations, such as pain, restricted range of motion, and mechanical impingement, rather than a visible deformity. Direct impingement with the glenoid labrum could lead to a limited range of movement [5].

Ravikanth et al. reported a similar case of osteochondroma arising from the left posterior humeral head within the joint capsule, causing restriction of the range of shoulder joint movements in a 61-year-old patient. The osteochondroma was completely excised surgically through a posterior axillary approach [6]. Padua et al. reported a very similar case to our case, where the osteochondroma of the left humeral head caused impingement on the glenoid bone and pain and restriction of joint movements in a 31-year-old male patient. Here, the lesion was also excised through the posterior capsular approach. The thickness of the cartilaginous cap was 1-3 mm [5].

Lee et al. reported a similar case of right humeral head intracapsular osteochondroma. Here, the patient had shoulder pain with restriction of joint movements. MRI of the shoulder demonstrated typical features of intracapsular osteochondroma with a cartilage thickness of less than a centimeter [7].

The radiological hallmark of osteochondroma is cortical and medullary continuity with the parent bone, best appreciated on CT or MRI. MRI is crucial for assessing the thin cartilage cap, which is characteristic of osteochondroma, detecting complications, and ruling out malignant transformation. A cartilage cap thickness of greater than 2 cm in adults or irregular surface morphology with marrow edema should raise suspicion for chondrosarcomatous transformation [8]. Mild reactive synovitis without significant secondary degenerative changes suggests relatively early intervention before chronic mechanical damage to the joint surfaces [7]. MRI is also crucial for evaluating the cartilage cap thickness and assessing any associated synovial or bursal reaction

The differential diagnoses of intracapsular bony lesions include synovial chondromatosis, dysplasia epiphysialis hemimelica (Trevors disease), para-articular chondroma, and intra-articular osteophyte. Synovial chondromatosis is benign metaplasia of synovium forming multiple cartilaginous nodules without stalk and medullary continuity. Dysplasia epiphysealis hemimelica (Trevors disease) is a benign asymmetrical bone overgrowth from the epiphysis seen in the knee or ankle. A para-articular chondroma is a soft tissue chondroma arising from the joint capsule with a distinct separation from the femur/tibia. An intra-articular osteophyte is a calcified/ossified body within the joint with no bony stalk/medullary continuity.

In the present cases, the well-defined medullary continuity, thin cartilage cap, absence of bone marrow edema, and the associated soft-tissue component favor the diagnosis of intracapsular osteochondroma. Table 1 summarizes the two cases presented in this report.

**Table 1:** Summary of the cases

	Case 1	Case 2
Age	49 years	14 years
Gender	Female	Male
Side	Left	Right
Site	Intracapsular shoulder joint	Intracapsular shoulder joint
Joint changes	Joint effusion (+)	Joint effusion (+)
Soft tissue component	No	No
Cartilage thickness	3 mm	2 mm
Management	Conservative management	Conservative management
Follow-up	The patient was doing better symptomatically.	The patient was doing better symptomatically.

In addition to occurring as solitary lesions, osteochondromas may be part of hereditary multiple exostosis (HME), also termed diaphyseal aclasis, which is an autosomal dominant skeletal dysplasia caused by variants in EXT1 and EXT2. They present as multiple osteochondromas, limb deformities, limb length discrepancy, and with a higher risk of developing secondary chondrosarcoma. Common sites include the humerus, hips, knee, and scapula. In such cases, a detailed family history, screening radiographs of other typical sites, and genetic counselling with long-term surveillance are advised [9].

Potential complications of osteochondromas include a fracture at the base of the pedunculated lesion, neurovascular compression, capsulitis, bursitis, and sarcomatous transformation [10].

Surgical excision is the treatment of choice, particularly in symptomatic cases or when the lesion restricts joint motion or demonstrates interval growth. Complete removal of the lesion, including the cartilaginous cap, minimizes the risk of recurrence [11]. Postoperative physiotherapy and early mobilization play a crucial role in restoring full joint mobility.

## Conclusions

Intracapsular osteochondroma of the proximal humerus is an extremely rare entity that can mimic other intra-articular pathologies, causing shoulder pain and restriction of movement. Detailed imaging evaluation with MRI, in addition to X-ray, is vital for accurate diagnosis and preoperative planning. Early surgical excision results in complete symptom relief, restoration of joint function, and prevention of degenerative changes.
